# Intradermal Delivery of Antigens Enhances Specific IgG and Diminishes IgE Production: Potential Use for Vaccination and Allergy Immunotherapy

**DOI:** 10.1371/journal.pone.0167952

**Published:** 2016-12-14

**Authors:** Takuwa Yasuda, Takehiro Ura, Masaru Taniguchi, Hisahiro Yoshida

**Affiliations:** 1 Laboratory for Immunogenetics, RIKEN Research Center for Integrative Medical Sciences (IMS), Yokohama, Kanagawa, Japan; 2 Hospital Company R&D Department, Terumo Corporation, Kanagawa, Japan; 3 Laboratory for Immune Regulation, RIKEN Research Center for Integrative Medical Sciences (IMS), Yokohama, Kanagawa, Japan; King's College London, UNITED KINGDOM

## Abstract

Skin is protected by a tough but flexible multilayered barrier and is a front line for immune responses against invading particles. For many years now, skin has been a tissue where certain vaccines are injected for the prevention of infectious disease, however, the detailed mechanisms of the skin immune response are not yet well understood. Using thin and small injection needles, we carefully injected OVA into a restricted region of mouse skin, i.e., intradermal (ID), and examined the antibody response in comparison with subcutaneous (SC) injection or epicutaneous patch administration of OVA. Epicutaneous patches induced a high IgE response against OVA, but IgG production was low. High IgG production was induced by both ID and SC injection, moreover, ID injection induced higher IgG production without any adjutants. Furthermore, OVA-specific IgE production was diminished by ID injection. We found that ID injection could efficiently stimulate skin resident DCs, drive Th1-biased conditions and diminish IgE production. The ID injection response was regulated by Langerin^+^ dermal DCs, because OVA was taken up mainly by these cells and, after transiently deleting them, the IgE response was no longer diminished and IgG1 production was enhanced. We also tested whether ID injection might be an effective allergy treatment by attempting to inhibit ongoing IgE production in mice with experimentally induced high serum IgE levels. Multiple ID injections of OVA were shown to prevent elevation of serum OVA-specific IgE after repeated allergen challenge. In contrast, SC OVA injection could only transiently inhibit the OVA-specific IgE production. These findings indicated that ID injection results in higher induction of antigen-specific IgG, and thus may be useful for vaccine delivery with little or no adjuvant components. Moreover, the observed diminishment of IgE and induction of Th1-biased immune responses suggest that ID may be a useful injection route for allergy immunotherapy.

## Introduction

Skin is an enormous organ that separates the outside world from our bodies. It is flexible, but also has a distinct barrier function to block penetration of external foreign particles and prevents excessive water loss [1, 2]. To perform these functions, our skin is composed of multiple layers, hair, stratum corneum, stratified epidermal cell layer, dermal connective tissue layer, sub-dermal, looser connective tissue layer, fat layer, and skin muscle. In addition to these layers, multiple structures such as nerve cells, blood vessels, lymphatic and immune cells are densely distributed in skin [3, 4]. As a result, skin plays a role as both a sensory organ and an immune organ.

As an immune organ, skin faces continuous challenge by a variety of foreign substances that can potentially invade the host. Some small chemicals can easily pass through the skin barrier [5]. On the other hand, microbes are normally repulsed by this barrier, but are able to enter into the epidermal or dermal layers via small lesions or epidermal turnover [6]. Finally, parasites can penetrate deeply into the skin as a result of bites by their vectors, such as mosquitoes or ticks, and induce distinct immune responses [7]. These various invaders that break through our skin barrier system are recognized and rejected by physical or immunological reactions in a manner appropriate for each of them.

For centuries, we have been using the skin immune system for disease prevention without much knowledge of its function. In 1796, Edward Jenner succeeded in preventing smallpox after vaccinating with the related cowpox virus by scratching virus-containing lesion material onto human skin. Since then, the skin has also been the most frequently used body region for vaccination against various pathogenic microbes [8], and inoculation of antigens into the skin dermal layer is currently considered a very efficient route for vaccination [9]. Intradermal (ID) vaccination is effective in inducing production of IgG antibodies specific for several viruses, such as influenza, B hepatitis, and rabies. Moreover it is also effective at inducing immune responses in elderly people and other low responder populations who usually did not respond well to other vaccination routes [10]. The Mantoux technique currently used for ID injection requires skilled medical workers and is difficult to perform successfully because it sometimes results in administration of the vaccine at an inappropriate depth [11].

In order to establish a reliable method to deliver vaccines into the dermal layer without any clinical skills, some groups developed ID injection devices. For example, an ID device with a 1.15 mm long needle pressed perpendicularly to the skin injects a very small amount of influenza vaccine without leaking and resulted in greater immunogenicity and safety compared to SC vaccination [12, 13].

As mentioned, the immune reaction against various foreign particles penetrating through the skin should be required. Therefore, it seems reasonable to suggest that the ID injection optimal route for vaccination. The precise mechanism of ID injection effectiveness in antibody production, however, has not been clarified.

Here we have examined the mechanism of ID injection-induced immunity by using a small, thin needle and compared it with SC injection and the epicutaneous patch. In mice immunized by the epicutaneous patch [14], serum antigen-specific IgE was significantly elevated but IgG was not. Antigen-specific IgG was induced more efficiently by ID injection than by SC injection or epicutaneous patch. Interestingly, antigen-specific and total IgE production was lower in ID than in SC injected mice. Therefore, the ID injection route was found to be effective both in inducing IgG production and in preventing IgE production in mice. By flow cytometry analysis of draining lymph node cells after antigen administration, we found that this effect was dependent on resident dermal dendritic cell populations, because it was lost after depletion of these cells by genetic manipulation [15].

Based on its efficacy in diminishing IgE production, we tested whether ID injection can contribute to the desensitization procedure currently used for allergy immunotherapy [16]. We first induced high serum antigen-specific IgE by epicutaneous patches, and then tried to prevent further IgE elevation after subsequent antigen challenge. ID injection prevented antigen-specific IgE elevation even when mice were challenged three times during half a year. In contrast, SC injection only prevented serum IgE elevation after the first challenge, failing after the second and third challenge.

Here we show the result in detail and discuss about the mechanism of immune reaction after ID antigen administration.

## Materials and Methods

### Animals

All experiments with mice were approved by the Animal Experiment Committee at RIKEN, Yokohama Institute and the Institutional Animal Care and Use Committee at Terumo. All mouse work was carried out in accordance with applicable guidelines and regulations. BALB/cA and C57BL/6J mice were purchased from CLEA Japan. Langerin-DTR mice were provided by B. Malissen. The mice were maintained under specific pathogen-free conditions and were held under controlled temperature (23°C ± 2°C) and humidity (50% ± 10%) with a 12-hour light-dark cycle in the RIKEN IMS animal facility or Terumo Corporation animal facility. Food and water were delivered ad libitum. For injection of antigen and sampling of blood, the mice were initially anesthetized with 2–4% isoflurane until they stopped making a movement. Then the mice were continuously maintained with 1–2% isoflurane. For sampling of tissue, mice were sacrificed by cervical dislocation. All efforts were made to minimize animal suffering.

### Injection and patch sensitization

Mice were injected with 0.2, 2 or 20 μg of OVA (fraction V; Sigma) in 2 μl saline via SC or ID route using a regimen shown in each figure. OVA was quantified the contamination of endotoxin by Chromogenic LAL Endotoxin Assay kit (GenScript). The endotoxin unit in OVA was 0.37 per ml. All the injection needles were supplied by Terumo and 26G x 1/2” and a 36G ID injection system were used for SC and ID injection, respectively. For epicutaneous sensitization, 200 μg of OVA in 200 μl saline was placed on a sterile gauze BAND-AID patch (Johnson & Johnson), which was secured to the back skin and covered with Transpore Surgical Tape (3M). Two days after the first sensitization, the adhesive bandage was removed, and OVA was applied topically on the shaved back. Every allergen sensitization was performed under anesthesia and the back hair was carefully shaved in order not to damage the skin surface. Blood samples were collected at 3 days before the first sensitization (day-3) and 18 days after the first sensitization (day 18). In some experiments, Langerin-DTR and control WT B6 mice were administered diphtheria toxin (DT) at 8.0 ng/g body weight via intraperitoneal injection one day before the OVA injection. In order to prevent the death of mice during sensitization, DT and OVA injections were performed 3 times at intervals of a week and blood samples were collected at 3 days before the first sensitization (day-3) and 18 days after the first sensitization (day 18).

### Enzyme-linked immunosorbent assay (ELISA)

Total IgG1, IgG2a, IgG2c levels were determined using an ELISA Quantitation Set (Bethyl). IgE and OVA-specific IgE levels were measured with an ELISA MAX Standard Set (Biolegend) and DS Mouse IgE ELISA (OVA) (DS Pharma Biomedical), respectively. For OVA-specific IgG1, IgG2a and IgG2c detection, serial diluted sera were sandwiched between plate-coated 1.0 μg OVA and horseradish peroxidase-conjugated anti-mouse IgG1 (Rat-mono, LO-MG1-2; AbD Serotec), IgG2a (Rabbit-poly; Invitrogen) or IgG2c antibodies (Goat-poly; Bethyl). The reactions were developed with 3, 3', 5, 5'- tetramethylbenzidine (TMB; KPL), and the absorbance was read by plate reader. Anti-ovalbumin IgG1 antibody (mouse-mono, 8C6; Thermo) and anti-denatured ovalbumin IgG2a antibody (mouse-mono, 6G2; Thermo) were used as reference antibodies. The relative concentration of OVA-specific IgG2c was compared by absorbance, because no anti-ovalbumin IgG2c antibody could be obtained.

### Flow cytometry

Mice were injected with 2 μg of OVA or Alexa Fluor 488 labeled OVA (Life Technologies) in 2 μl saline by SC or ID route. A quantification of endotoxin in Alexa488 labeled OVA was performed by Chromogenic LAL Endotoxin Assay kit. The endotoxin unit was 0.54 per ml. Skin draining LNs (axillary and brachial) were isolated 24 hrs after the injection of Alexa488 labeled OVA, and dissociated with 0.167 mg/ml Collagenase I (Sigma) and 0.02 mg/ml DNase I (Roche) into single cell suspensions for 30 min at 37°C. Washing with PBS, cells were stained with PE or PerCP-Cy5.5-anti CD103 (BioLegend; clone 2E7), PE-Cy7-anti CD11c (BioLegend; clone N418), BV421-anti CD326 (BD Biosciences; clone G8.8), BV510-anti MHC class II (BioLegend; clone M5/114.15.2) and anti CD301b (BioLegend; clone URA-1). The CD301b antibody was labeled in house with a CF647 labeling Kit (Biotium). 7AAD^+^ cells were excluded as dead cells. For intracellular Langerin staining, cells were stained with PE-anti CD207 (eBioscience; clone eBioL31) using Foxp3/Transcription Factor Staining Buffer Set (eBioscience). Dead cell exclusion was performed using Fixable Viability Dye efluor 780 (eBioscience). Stained cells were analyzed by flow cytometry (FACSAria; BD Biosciences) and data were analyzed using FlowJo software (TreeStar). Example of gating strategy was shown in S1 Fig. Briefly, white blood cells were gated based on forward scatter and side scatter. Doublet cells were eliminated by FSC-A versus FSC-H and SSC-A versus SSC-H gating. 7AAD^+^ cells were excluded as dead cells. OVA uptaken DC was regarded as Alexa488^+^/MHC-class II^high^ population. After gating on the Alexa488^+^/MHC-class II^high^ cells, three DC subsets that of CD301b^+^ dDC, LC and CD103^+^ dDC were evaluated.

### Ultrasound echographic image

BALB/cA mice were sacrificed and back hair was carefully shaved, and then, 2 μl of India ink was injected via SC or ID route. Immediately after injection, the ultrasound echographic images of injection site were obtained by ultrasound biomicroscope NP60R-UBM (Paradigm medical industries)

### Histological and immunohistological analysis

The ink injected skin samples were cut out and fixed with 4% paraformaldehyde, embedded in polyester wax (VWR International), and sectioned for hematoxylin and eosin (H&E) staining observation. To detect Langerin^+^ cells, the back skin was removed at 24 hours after DT injection, and frozen skin specimens were sectioned and fixed by microwave irradiation and methanol. After blockade with 3% BSA, 2% skim milk, 0.1% Triton X100 in PBS, slides were stained with Langerin (Rabbit IgG poly; IMGENEX) antibody in 2% skim milk, 0.1% Triton X100 in PBS, followed by incubation with FITC-conjugated swine anti-rabbit Igs (DAKO) secondary antibody. Slides were counterstained with DAPI (Vector Laboratories).

### Statistics

Statistical significance was calculated by the Student’s t-tests or by the Bonferroni’s test following 1-way ANOVA for multiple comparisons. *P* values of 0.05 or less were considered significant.

## Results

### Different routes of skin immunization result in different antibody responses

**Fig 1:**
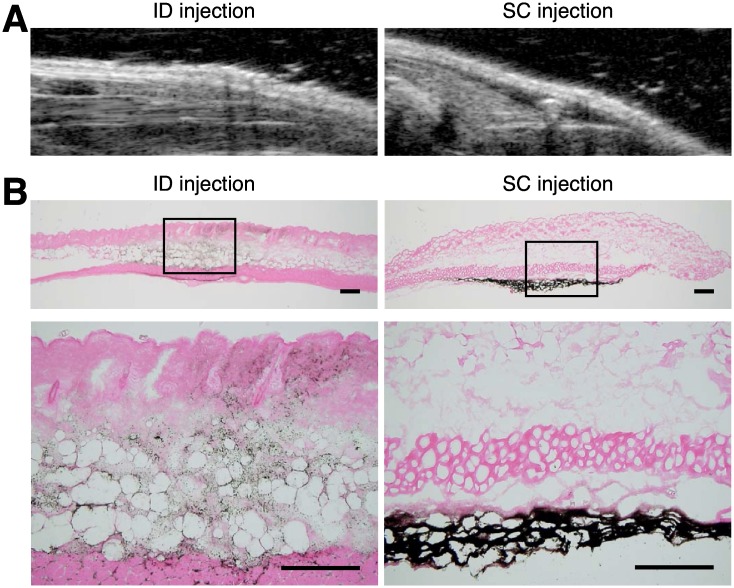
Distinct distribution of injected fluid via intradermal or subcutaneous route. (A) The ultrasound echographic images of injection site of skin were obtained immediately after 2 μl of India ink injected with 36G ID injection system for ID or 26G injection needle for SC. (B) Photomicrographs of skin histological sections. Immediately after injection via the ID or SC route, injected skin region was cut out and performed H&E staining. Lower panels are magnified view of boxed region in upper panels. Scale bar, 100 μm.

In order to understand the effect of ID injection on the immune response, we immunized BALB/c mice with 1 mg/ml ovalbumin (OVA). For precise injection of OVA into the dermal region of mice, we used special small thin injection needle supplied by Terumo (36G ID injection system). Representative ultrasound echographic and histological images of injection site were shown in Fig 1. Using this ID device, injected fluid was distributed in the skin dermal and/or epidermal region. In contrast, distribution pattern of injected fluid by SC injection performed with a 26G injection needle was absent from dermal or epidermal region, different from that of ID device.

**Fig 2:**
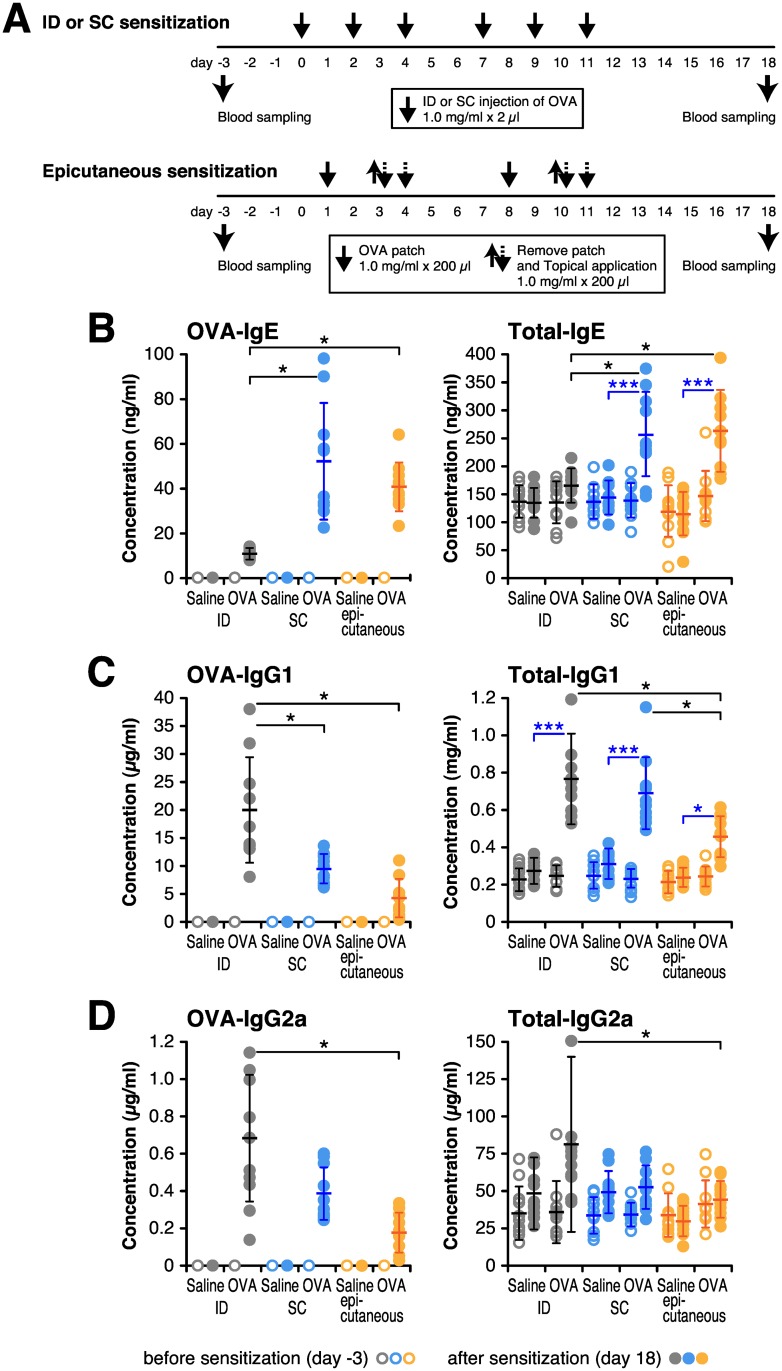
Intradermal antigen injection preferentially diminishes IgE antibody formation. BALB/c mice continuously received 6 times by intradermal (ID) or subcutaneous (SC) injection with 2 μg of OVA in 2 μl saline, or by patch and topical application (epicutaneous) with 200 μg of OVA in 200 μl saline. Blood samples were collected at 3 days before the first sensitization (day-3) and 18 days after the first sensitization (day 18). (A) Timeline for sensitization and blood sampling. (B–D) Concentrations of OVA-specific and total serum IgE (B), IgG1 (C) and IgG2a (D) were determined by ELISA. Each circle represents the concentration of individual 10 mice, and bar shows the mean ± SD. *P < 0.05, ***P < 0.001.

To compare the antibody responses of different routes, the same amount (2 μl) of OVA was administered by ID and SC route. Epicutaneous sensitization was done using a gauze patch on the shaved back skin with 200 μl of OVA, followed by painting 200 μl of the same solution on the next day. The injection and blood sampling schedule is shown in Fig 2A.

OVA-specific and total immunoglobulin levels were determined as IgE, IgG1 and IgG2a subclasses before and after OVA administration. Though it is difficult to certify that the amount of OVA introduced by epicutaneous immunization, the concentration of OVA-specific and total IgE was elevated to a comparable level by SC or epicutaneous antigen administration, but was significantly less in ID injected mice (Fig 2B). In contrast, the concentration of OVA-specific and total IgG1 and IgG2a was the highest in ID injected mice and lowest in epicutaneous sensitized mice (Fig 2C and 2D). These results indicated that the antibody response could be modified depending on the injection route.

### ID injection enhances antigen-specific IgG response with only a small amount of antigen

**Fig 3:**
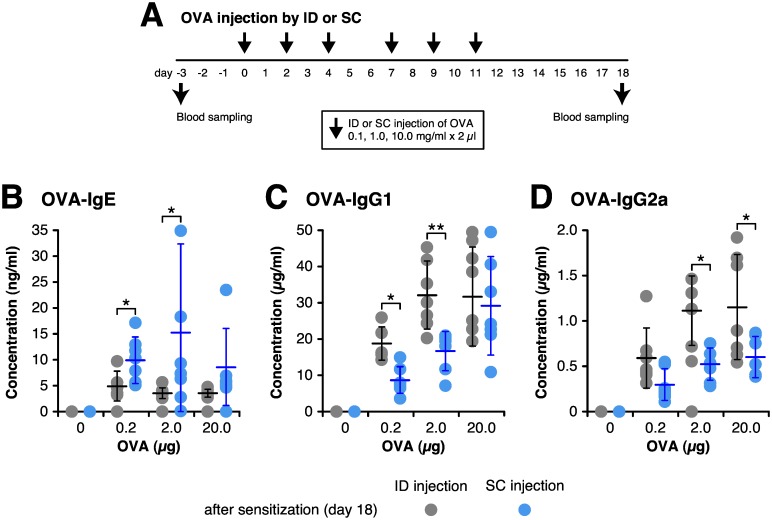
Intradermal injection efficiently induces antigen-specific IgG production. BALB/c mice continuously received 6 times by intradermal (ID) or subcutaneous (SC) injection with three different doses, either 0.2, 2 or 20 μg of OVA in 2 μl saline. Blood samples were collected at 3 days before the first sensitization (day-3) and 18 days after the first sensitization (day 18). (A) Timeline for injection and blood sampling. (B–D) Concentrations of OVA-specific serum IgE (B), IgG1 (C) and IgG2a (D) were determined by ELISA. Each circle represents the concentration of individual 7 mice, and bar shows the mean ± SD. *P < 0.05, ***P < 0.001.

Since ID injection induced IgG1 and IgG2a production more efficiently than SC or epicutaneous administration, we examined whether ID injection can induce an immune response with a lower dose of antigen compared to SC injection. OVA was set at 0.1, 1, or 10 mg/ml concentration in saline and 2 μl of each solution was injected repeatedly, then the serum immunoglobulin levels were examined (Fig 3A). Before sensitization, no OVA-specific immunoglobulins were detected. The concentration of OVA-specific and total IgE after ID or SC injection was comparable among the three OVA concentrations, although the IgE concentration in the ID group was always lower than in the SC group (Fig 3B and S2A Fig). ID injection induced high OVA-specific IgG1 and IgG2a titers using only a small amount (0.2 μg) of antigen. OVA-specific IgG1 and IgG2a levels induced by ID injection of 0.2 μg of OVA were comparable to those induced by SC injection of 2 μg of OVA (Fig 3C and 3D, S2B and S2C Fig), therefore ID injection was approximately 10 fold more effective than SC injection. However at high OVA dose (20 μg), serum IgG levels was comparable between ID and SC injection. In our injection protocol, the total serum IgG2a level was unaffected by the injection route at any OVA injection dose. These studies demonstrate that ID injection enhances antigen-specific IgG response after immunization with only a small amount of antigen.

### The migrated dendritic cells in draining lymph nodes is different after ID and SC injection

The observed differences in immunoglobulin production after ID and SC injection suggested differences in the cells participating in the immune response. Since the dendritic cells (DC) localized in the skin are reported to be different at different skin tissue depths [15, 17], we isolated draining lymph nodes (LNs) 24 hrs after injection, which reaches its peak of migrated DCs into LNs from peripheral site (S3 Fig), and examined whether DCs that had taken up antigen and migrated to draining lymph nodes (LNs) are different after ID and SC injection.

**Fig 4:**
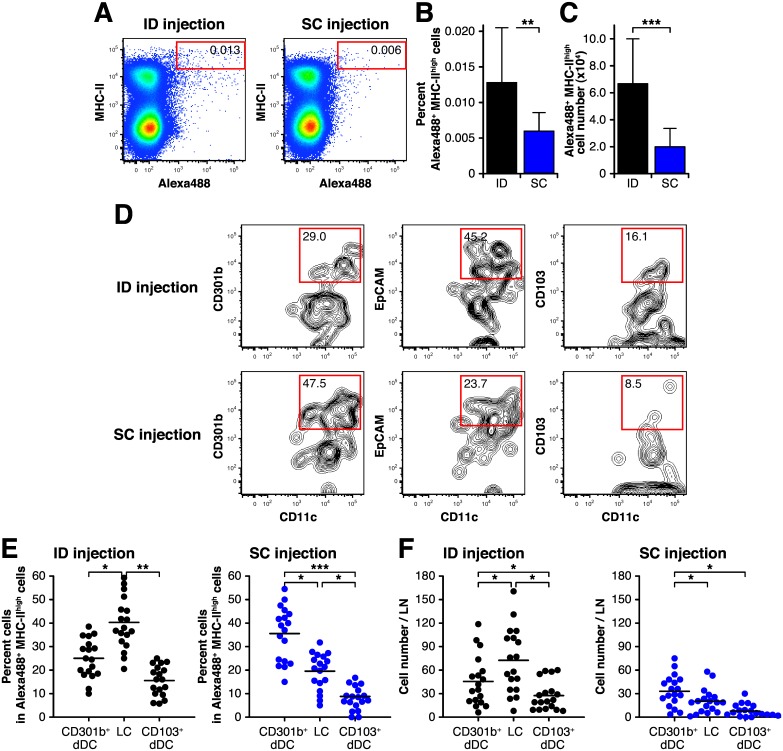
The dominant DC subset that migrates into the draining LN depends on the route of immunization. BALB/c mice were injected with Alexa488 labeled OVA via the intradermal (ID) or subcutaneous (SC) route and draining LNs were harvested 24 hr after the injection. Analysis of Alexa488^+^ cells in draining LN was performed by flow cytometry. (A) Representative FACS plots of LN cells from ID or SC injected mice. The percentages of Alexa488^+^/MHC-II^high^ cells in each plot are indicated. (B and C) The percentage (B) and absolute number (C) of Alexa488^+^/MHC-II^high^ cells in draining LN cells from ID or SC injected mice. Each bar shows the mean ± SD of 10 mice per group. **P < 0.01 and ***P < 0.001. (D) Representative FACS plots showing cells gated on Alexa488^+^/MHC-II^high^ LN cells from ID or SC injected mice stained for the indicated markers. The percentages of CD301b^+^CD11c^+^ (CD301b^+^ dDC), EpCAM^+^CD11c^+^ (LC) and CD103^+^CD11c^+^ (CD103^+^ dDC) cells in each plot are indicated. (E and F) Each circle represents the percentage of each DC subset among Alexa488^+^/MHC-II^high^ cells (E) and total cell number of each DC subset in draining LN (F) of individual mice, and horizontal bar indicates the mean. The experiments were independently performed 18 times. *P < 0.05, **P < 0.01, ***P < 0.001.

To accomplish this analysis, we injected Alexa488 labeled OVA and examined its distribution in draining LNs by flow cytometry. The MHC-class II^high^/Alexa488^+^ DC population was higher in ID versus SC immunized mice both in proportion (Fig 4A and 4B) and absolute number (Fig 4C). After gating on the Alexa488^+^/MHC-class II^high^ population of draining LN cells, we analyzed surface marker expression to distinguish the three DC subsets. The first subset is a CD301b^+^ dermal dendritic cell (dDC) that was recently reported to play a pivotal role in Th2-biased immune reactions in mouse skin [18, 19]. The second subset is the Langerhans cell (LC), identified by cell surface expression of EpCAM. The third subset expresses cell surface CD103 and is reported to participate in Th1 immune responses [20]. This subset was reported to be almost the same as the Langerin^+^ dDCs. In draining LN of ID injected mice, the frequency of LCs was highest and comprised 45% of the Alexa488^+^/MHC-class II^high^ population, followed in order by CD301b^+^ dDC and CD103^+^ dDC (Fig 4D, 4E and 4F). By contrast, the levels of CD301^+^ dDC were highest and accounted for about half the proportion of Alexa488^+^/MHC-class II^high^ cells in draining LN of SC injected mice, followed in order by LC and CD103^+^ dDC.

### Langerin^+^ DC in dermis contributes to the ID-specific immune response

Langerin is a cell surface molecule expressed by Langerhans cells in the epidermis and a subset of dDCs and both of these populations were reported to play important roles in skin immunity [13].

**Fig 5:**
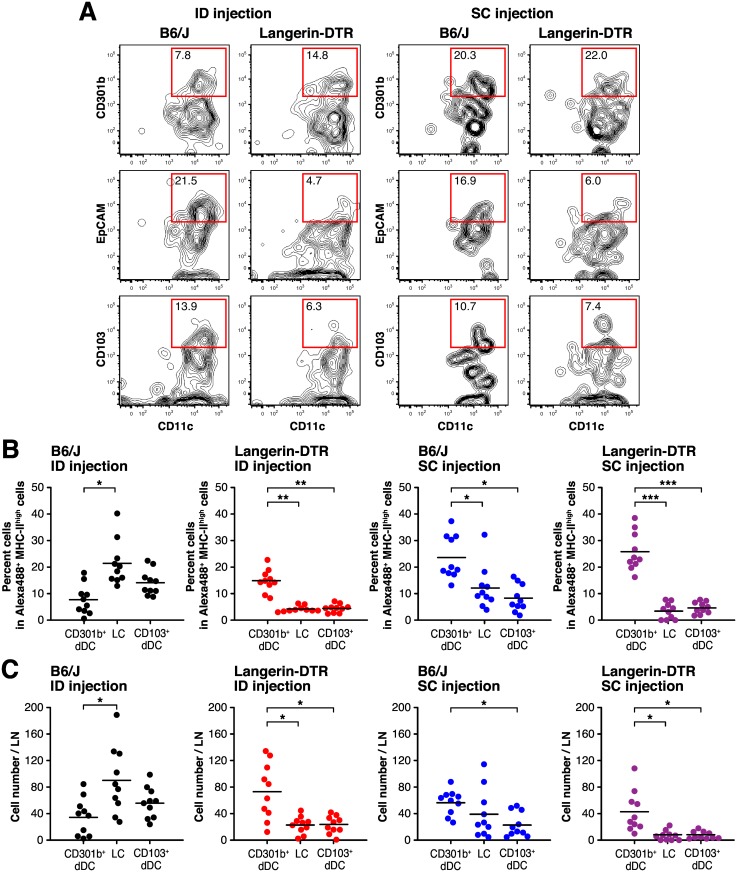
Langerin^+^ cells that migrate into the draining LN are efficiently decreased by DT injection in Langerin-DTR mice. (A) B6/J and Langerin-DTR (B6/J background) mice were administered DT via intraperitoneal injection one day before each OVA injection. Mice were injected with Alexa488 labeled OVA via the intradermal (ID) or subcutaneous (SC) route and draining LNs were harvested 24 hrs later. (A) Representative FACS plots showing cells gated on Alexa488^+^/MHC-II^high^ cells from ID or SC injected mice. The percentages of CD301b^+^CD11c^+^ (CD301b^+^ dDC), EpCAM^+^CD11c^+^ (LC) and CD103^+^CD11c^+^ (CD103^+^ dDC) cells in each plot are shown. (B and C) Each circle represents the percentage of each DC subset among Alexa488^+^/MHC-II^high^ cells (B) and total cell number of each DC subset in draining LN (C) of individual mice, and horizontal bar shows the mean. The experiments were independently performed 10 times. *P < 0.05, **P < 0.01, ***P < 0.001.

Since Langerin-expressing cell numbers were higher in ID than in SC immunize mice, we assessed their function by depleting them in mice transgenic for the diphtheria toxin (DT) receptor gene controlled by the Langerin gene promoter. As preliminary experiment, we confirmed the depletion of skin resident Langerin^+^ cells within 24 hrs after injection of DT at 8.0 ng/g body weight (S4A Fig). We also confirmed the depletion of Langerin^+^ cells in draining LN at 24 hrs after OVA injection (S4B and S4C Fig). Thus we performed DT injection 24 hrs before Alexa488 labeled OVA injection and the three DC subsets identified by CD301b, EpCAM or CD103 cell surface expression were analyzed by flow cytometry. Representative FACS plots of Langerin-DTR mice on the C57BL6/J (B6/J) background and wild-type B6/J mice in combination with ID or SC injection of Alexa488 labeled OVA are shown in Fig 5A. The percentage of each DC subset among Alexa488^+^/MHC-class II^high^ cells and cell number in draining LN of each DC subset is plotted in Fig 5B and 5C. DT injection efficiently decreased LC and CD103^+^ dDC subsets of both ID and SC injected Langerin-DTR mice. When we injected Alexa488 conjugated OVA by the ID route, CD301b^+^ dDC went from being the smallest subset in B6/J mice to become the most abundant subset in Langerin-DTR mice. In SC injected B6/J and Langerin-DTR mice, depletion of Langerin^+^ cell subsets did not affect the proportion of CD301b^+^ dDC.

**Fig 6:**
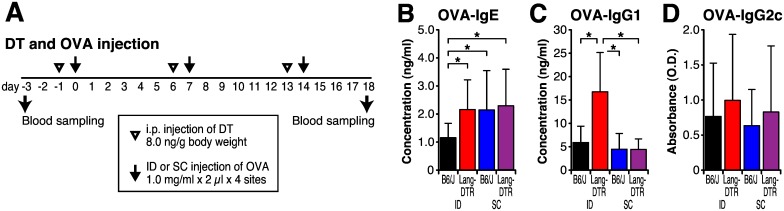
Langerin positive cells are required for diminishment of IgE production. B6/J and Langerin-DTR mice continuously received 3 times of DT and OVA by intradermal (ID) or subcutaneous (SC) injection with 2 μg of OVA in 2 μl saline. Blood samples were collected at 3 days before the first sensitization (day -3) and 18 days after the first sensitization (day 18). (A) Timeline for injection and blood sampling. (B–D) Serum concentrations of OVA-specific IgE (E), IgG1 (F) and IgG2a (G) were determined by ELISA. Each bar shows the mean ± SD of 8 mice per group. *P < 0.05.

Next, we examined whether the distribution of antibody isotypes was affected by depletion of Langerin^+^ cells. In our preliminary experiment, a week interval is requisite for preventing the death of mice during multiple DT injection. Thus we determined optimal DT and OVA injection timelines as shown Fig 6A. On this timelines, ID injected B6/J mice induced significantly lower OVA-specific IgE compared to SC. Compared to wild-type B6/J mice, the diminish effect of OVA-specific IgE production was lost in Langerin-DTR mice which depleted Langerin^+^ cells (Fig 6B). This depletion also increased the OVA specific-IgG1 levels in ID injected Langerin-DTR mice (Fig 6C), whereas the concentration of IgG2a (Fig 6D). These results indicate that the diminished IgE production seen after ID immunization is due to suppression of the response rather than, for example, that ID immunization does not provide the signals necessary for production. The results also show that Langerin^+^ cells are required for diminishment of IgE production after ID injection and may also play a role in IgG1 production.

### ID injection inhibits long-term IgE elevation and is a promising route for allergy immunotherapy

Desensitization by injecting small amounts of the allergen is often used clinically for the treatment of allergic disease [21]. This treatment is usually performed by SC injection. Since our results indicated that ID injection could diminish antigen-specific IgE production, we have tested the potential efficacy of intradermal allergy immunotherapy.

**Fig 7:**
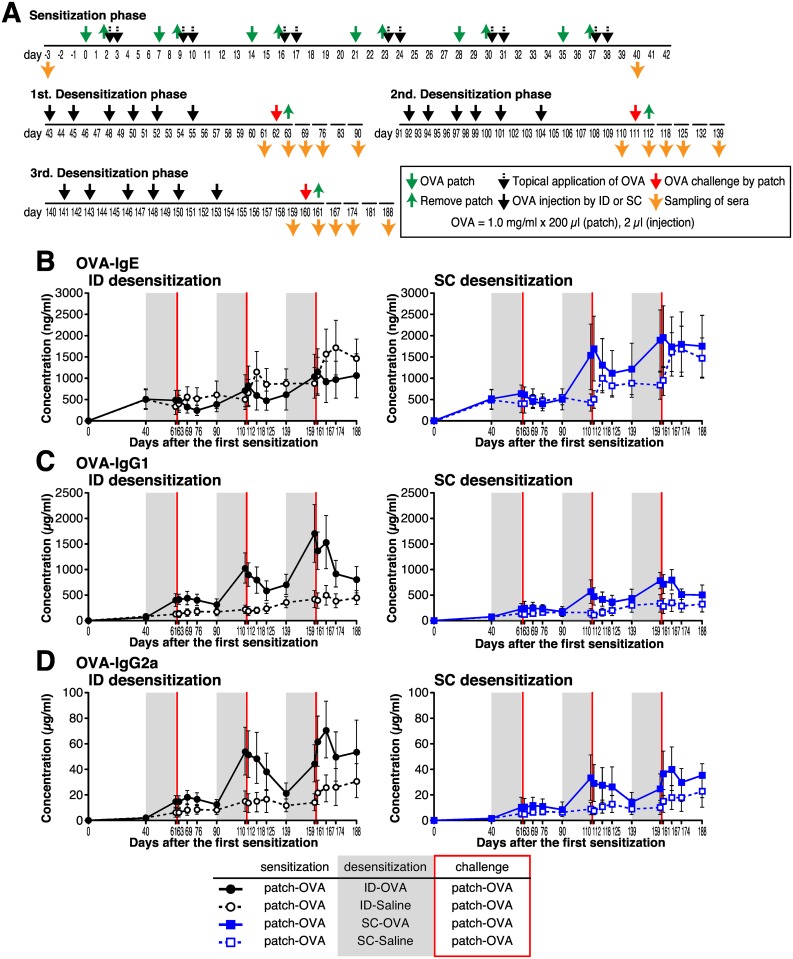
Repetitive desensitization via the intradermal route inhibits antigen-specific IgE elevation and enhances antigen-specific IgG production for the long-term. (A) Timeline for sensitization, desensitization and blood sampling. BALB/c mice were used in this experiment. (B–D) Serum concentrations of OVA-specific IgE (B), IgG1 (C) and IgG2a (D) were determined by ELISA. Data are the mean ± SD of 15 mice per group.

For these experiments, we sensitized mice to make high levels of serum OVA-specific IgE and then tested them for application of the desensitization phase. In the sensitization phase, BALB/c mice were repetitively stimulated with OVA using epicutaneous patches on their back skin for 6 weeks (Fig 7A) [22]. For the desensitization phase, mice were injected 6 times with OVA by ID or SC routes, and then an OVA patch challenge was performed. Considering the typical seasonal allergic disease treatment, such as for Japanese cedar pollen allergy [23], 30 days after the final OVA challenge we resumed desensitization and OVA patch challenge. We conducted the desensitization phase three times for a half a year in the same mice. As a control for this allergy immunotherapy model, we injected the same volume of saline (2 μl) by ID or SC route in the desensitization phase and then challenged 3 times with an OVA patch.

Desensitization by ID OVA injection clearly blocked the increase in serum OVA-specific and total IgE induced by OVA patch challenge in all three desensitization phases (Fig 7B and S4A Fig solid black line). In contrast, desensitization by SC injection was only effective at the first challenge and the OVA-specific and total IgE levels gradually increased after repetitive injections (Fig 7B and S5A Fig solid blue line). On the day after OVA patch challenge, there was a rapid increase in OVA-specific IgE in the control mice, but this was rarely observed in OVA desensitized mice. These results demonstrate that continual allergen injection by the ID route results in desensitization and long-term inhibition of IgE production.

In contrast to the inhibition of IgE elevation in the desensitization phase, the serum OVA-specific IgG1 and IgG2a levels were gradually elevated in both ID and SC routes of OVA injection. Comparing them, ID OVA injection induced much more IgG production than did SC injection (Fig 7C and 7D solid black and blue lines). Total serum IgG1 and OVA-specific IgG1 levels showed similar behavior (Fig 7C, S5B Fig), but total IgG2a levels were unaffected with desensitization by ID or SC injection under this experimental setting (Fig 7D, S5C Fig). These results indicate that the careful injection of antigen into the dermal region is highly effective for the long-term allergy immunotherapy.

## Discussion

In this study, we found that antigen delivery by ID injection can induce better antigen-specific IgG production than SC injection, and that antigen-specific IgE production is diminished by ID injection. These effects were seen even with a very small amount of antigen without addition of any specific adjuvant such as Alum or poly I:C.

We found that Langerin^+^ DCs were more plentiful in ID antigen-injected mice than in SC injected mice. The DT receptor expressing cells depletion experiments suggest that Langerin^+^ DCs contribute to the ID-specific immune response. Since Langerin^+^ dDC and LC in epidermis were reported to play important roles in Th1-biased immunity [24], the present results indicate that ID antigen injection efficiently stimulates those DCs to drive the skin immune response to a Th1-biased condition and enhances IgG production. The relative increase of Langerin^+^ DCs in draining LN may also contribute to diminishment of IgE production or Th2-biased immune responses. In contrast, CD301b^+^ dDCs, which have been reported to play important roles in Th2-biased immunity [18, 19], were highest in SC injected mice among the three DC subsets we tested, suggesting that CD301b^+^ dDC subset may contribute to IgE production. In addition, antigen sensitization via the epidermal surface by an epicutaneous patch significantly elevated the levels of serum IgE. These results indicate that the difference in skin depth where the antigen is delivered can induce different immune responses.

Why does the skin depth where antigen is delivered result in quite different immune responses? This result may reflect the history of skin immune system development for protection against invasion by different foreign substances, which destroy the skin barrier system and enter into the skin in different ways. Small molecules under 50 kDa molecular weight, such as chemicals and haptens, can easily penetrate through the stratum corneum and promptly reach the stratified keratinocyte cell layer. However, for larger particles, such as bacteria, invasion is normally blocked by the skin barrier system. Physically the stratum corneum is a strong multilayered barrier to bacterial entrance, and even if they can penetrate through the corneum and enter into the epidermal layer, their proliferation is usually blocked by low pH and the response of innate immune cells, in combination with rapid proliferation and turnover of keratinocyte cells. But if bacteria enter into the dermal layer just beneath the keratinocyte basal layer via a skin wound, they can proliferate to induce pathological changes in skin tissue [25]. In other words, the skin immune system must have a dermal immune system for protection against bacterial invasion into the dermal layer. This could be the reason why ID antigen injection enhanced production of IgG but not IgE. On the other hand, parasites are far larger than bacteria. For example, in the case of biting by ticks and mosquitoes, bacteria are often present in their saliva or on the surface of the stinger, which can easily penetrate the epidermis and dermis to reach the SC fat layer or skin muscle layer [26]. This may explain why SC antigen injection resulted in elevation of both IgG and IgE. In addition, some parasites such as house dust mites attach onto the skin surface to eat the desquamated corneal layer or sebum [27]. They usually grab the hair shaft or epidermal layer surface and do not invade into the dermal region. This may explain why epicutaneous antigen sensitization often resulted in serum IgE elevation rather than IgG production.

Therefore, if one could deliver the antigen specifically into the intradermal space, there would be higher IgG production under Th1-biased immune conditions, with suppressed Th2-biased immune responses. This dermal specific Th1-biased immunity is regulated by Langerin^+^ DCs localized in the dermis. From this point of view, ID injection should be very suitable for vaccination against microbes. It contributes to enhancement of immunogenicity and improves vaccine efficacy, especially in individuals with low antibody response. However our experimental data of antibody responses demonstrated that the degree of individual variation is comparable between ID and SC. This indicated that regional antigen delivery is unrelated to variability of antibody response.

In several previous reports, use of an ID device was shown to decrease the volume of viral antigen required [8], since IgG production was more efficient than with SC injection, and it was also reported that ID vaccination is effective with little or no adjuvant components. Currently, most human vaccines contain some kind of adjuvant. In most cases, this small amount of adjuvant is not harmful, however, some of the serious adverse reactions following vaccination are thought to be induced by adjuvant co-injection [28]. The incidence of such adverse reactions is low enough that it has been ignored based on economic considerations, however, if a small amount of a purified antigen without any adjuvant can be used as a vaccine, we can entirely avoid any problems induced by adjuvant co-administration [29].

Another concern about vaccination is the anaphylactic reaction. This allergic reaction against the antigen is usually mediated by binding of antigen-specific IgE and stimulation of mast cells or basophils, and can be fatal [30, 31]. Since antigen administration by the ID route can diminish antigen-specific IgE production, it should be safer in terms of avoiding anaphylactic shock than other vaccination methods.

Focusing on this lower IgE production after ID antigen delivery, we attempted to use ID injection for allergy immunotherapy model and observed a clear inhibitory effect on IgE production. Even after three rounds of desensitization treatment and challenge in the same mice, desensitization by ID injection could inhibit the elevation of serum IgE titer during half a year period. In contrast, using conventional desensitization by SC injection, we could not continuously inhibit IgE production, although the initial effect was comparable to desensitization by ID injection. These results suggest that the injection of allergen into the dermal region is more suitable for allergy immunotherapy, because this procedure typically requires repetitive administration of small amounts of allergen anyway [23]. Recently many route for allergy immunotherapy has been attempted such as subcutaneous, oral, sublingual [32], and epicutaneous patch [33]. Among them, desensitization by ID has been assessed in humans by Rotiroti et al whose results were similar to ours. They explained that repeated ID injection with low-dose grass pollen extract induced higher IgG and block IgE-allergen complex binding to B cells [16]. Although the mechanism of allergy desensitaization immunotherapy is not fully understood, ID may have a potential for improving the result via injection route by means of its unique immune responses affecting the skin resident LCs and transporting allergen directly LNs.

What are the mechanisms that diminish IgE production after ID antigen administration? In allergic reactions, multiple steps are thought to participate in enhanced IgE production [34]. First, antigen-presenting cells such as DCs capture allergens in epithelial tissue exposed to the environment. Second, these antigen-presenting cells interact with helper T cells in the nearest peripheral lymphoid tissue and induce differentiation and proliferation of Th2 type helper T cells. Third, the Th2 cells induce activation of allergen-specific B cells by direct cell contact and by secretion of cytokines such as IL-4, which also promotes class switching to IgE. Finally, these B cells differentiate into long-lived plasma cells secreting allergen-specific IgE for long periods [35]. Since the latter three steps take place in the peripheral lymphoid tissue, the IgE inhibitory mechanism induced by ID injection must occur during the first step. Strong candidate cells are antigen-presenting cells such as DCs and macrophages [19].

Therefore, we examined differences in surface marker expression on MHC-class II^high^ DCs in the draining LN after ID and SC injection and found that ID injection was better than SC injection at increasing the number of such DCs. Thus, it is plausible that simply the difference in total DC numbers in draining LN is the reason why IgG production is enhanced by ID antigen injection. On the other hand, we found that the MHC-class II^high^ cell subsets were different between ID and SC injected mice. In particular, Langerin^+^ cells were higher in ID injected mice than in SC injected mice. Langerin^+^ cells might be responsible for diminishment of IgE production, as depletion of Langerin^+^ cells restored IgE production that had diminished in ID injected mice. It is interesting for us to know how Langerin^+^ cells that stimulated for a long period act on IgE production. Although contribution of Langerin^+^ cells in allergy immunotherapy model is unclear, as we preliminarily checked that the repeated DT injection was harmful for mice, Langerin^+^ cells assumed to contribute and affect immune responses also during the long-term protocol. Additionally, when considering the diminished IgE production after ID antigen administration, we may have to take the difference of genetic background of mice into account. In the present study, OVA specific IgE titers of BALB/c mice were higher than those of B6/J mice and ID injection obviously diminished the IgE production. Given that Th2-biased BALB/c mice have been reported to produce much more IgE responded to allergic sensitization, compared with B6/J mice [36], mouse strain differences are likely to affect magnitude of the effect of ID injection.

Since the establishment of vaccination by Edward Jenner at the end of 18^th^ century, the mechanism of immune responses to vaccines has been extensively investigated and effective adjuvants have been discovered and used to obtain the appropriate concentration of serum immunoglobulins as soon as possible after vaccination. However, in order to avoid adverse effects such as IgE-induced anaphylactic shock, more efficient and safe adjuvants are still being investigated. Similarly, to achieve the maximum effect, we need to investigate and select the best route of immunization, such as transdermal, intradermal or subcutaneous. The ID route is effective in terms of diminishing IgE production, as presented in this study, although it requires certain technical skills to inoculate antigens into the thin dermal layer. A device that is easy to handle and designed to deliver antigen into a restricted region of the skin will be helpful for safe and secure vaccination by the ID route. In this point of view, various approaches have performed to penetrate vaccine across the skin such as solid multi-needles systems which possess well-controlled needles length, hollow single needle systems aim to reliable injection [12, 13], transdermal patches in order to self-administration of vaccines [37]. Biodegradable polymeric technology enables novel vaccines with antigen-loaded dissolvable microneedles [38, 39]. However, as we indicated opposite effect of IgG and IgE production between epicutaneous and intradermal administration, immunogenicity of vaccine could be different even among these similar skin delivery devices. It might depend on reliability of injection to avoid the vaccine leakage on the skin surface [40], target skin depth [41] and vaccine form [42].

At least two independent mechanisms for enhancing immunogenicity of intradermal vaccine have been suggested. 1) ID vaccine efficiently stimulates skin resident cell subsets. Once exposed to antigens, the cells promptly migrated into draining LNs and enhancing antigen-specific responses [43, 44]. 2) ID could immediately transport vaccine to antigen-presenting cells in the lymph nodes via lymphatic vessels [45]. In this our experiments, we showed the frequency and number of DC subsets in draining LNs. However the degree of contribution of this short time lymphatic drainage is difficult to argue. Lymph draining flow rate is considered to relate to interstitial fluid pressure of skin [46]. Consistent with this, Tozuka et al, reported that injection volume of ID affects vaccine distribution via lymphatic flow [47]. Thus low volume ID OVA injection as we examined here, preferably resides inside the skin immediately after the injection.

In this study, we have found that simply injecting a small amount of antigen into dermal region can induce antigen-specific IgG efficiently and can also diminish IgE production. We think that ID injection has great potential for application in allergy immunotherapy and could be epoch making in the history of vaccination.

## Supporting Information

S1 Fig The gating strategy for the flow cytometry experiments. Draining LNs were isolated from mice injected with Alexa488 labeled OVA via the intradermal (ID) or subcutaneous (SC) route and analyzed by flow cytometry. Dot plots show the gating strategy used in this study. DCs that migrated into the draining LN were analyzed on total doublet^-^, live (7AAD^-^), Alexa488^+^, MHC-II^high^, CD11c^+^ cells. The three DC subsets were distinguished by surface marker (CD301b, EpCAM, CD103) expression.
(TIF)

S2 Fig Effect of intradermal injection on total IgE and IgG production. (A–C) BALB/c mice received OVA by intradermal (ID) or subcutaneous (SC) injection. Concentrations of total serum IgE (**A**), IgG1 (**B**) and IgG2a (**C**) were determined by ELISA. Each circle represents the concentration of individual 7 mice, and bar shows the mean ± SD. *P < 0.05, ***P < 0.001.
(TIF)

S3 Fig The kinetics of migrated dendritic cells in draining LN after ID or SC OVA injection. BALB/c mice were injected with OVA via the intradermal (ID) or subcutaneous (SC) route and draining LNs were harvested 24, 48 or 96 hrs after the injection. Cells in draining LNs were analyzed by flow cytometry. (A–D) The percentage (A and C) and absolute number (B and D) of CD11c^+^MHC-II^high^ cells (A and B) or Langerin^+^ cells (C and D) within the total doublet^-^, live cells in the draining LN from ID or SC injected mice. Each bar shows the mean ± SD of 6–8 mice per group. *P < 0.05, **P < 0.01 and ***P < 0.001.
(TIF)

S4 Fig Langerin^+^ cells are depleted from skin and draining lymph node by DT injection in Langerin-DTR mice. (A) Frozen skin sections of B6/J and Langerin-DTR mice 24 hr after DT injection were stained with anti-Langerin (green), and nuclei were counterstained with DAPI (blue). Scale bar, 100 μm. (B and C) B6/J and Langerin-DTR mice were administered DT via intraperitoneal injection one day before each OVA injection. Mice were injected with OVA via the intradermal (ID) route and draining LNs were harvested 24 hrs later. FACS plots showing cells gated on total LN cells (B) and gated on CD11c^+^ cells (C) from WT and Langerin-DTR mice. Draining LN cells were stained for the indicated markers. The percentages of Langerin^+^ cells among total LN cells in each plot are shown.
(TIF)

S5 Fig Effect of repetitive desensitization via the intradermal route on total IgE and IgG production. (A–C) Serum concentrations of total IgE (**A**), IgG1 (**B**) and IgG2a (**C**) were determined by ELISA. BALB/c mice were used in this experiment, which was performed as shown in Fig 7A. Data are the mean ± SD of 15 mice per group.
(TIF)
