# Supplementary figures and images for: Intradermal Delivery of Antigens Enhances Specific IgG and Diminishes IgE Production: Potential Use for Vaccination and Allergy Immunotherapy
Source: PLoS One. 2016 Dec 14;11(12):e0167952. doi: 10.1371/journal.pone.0167952 (PMC5156430; doi:10.1371/journal.pone.0167952)

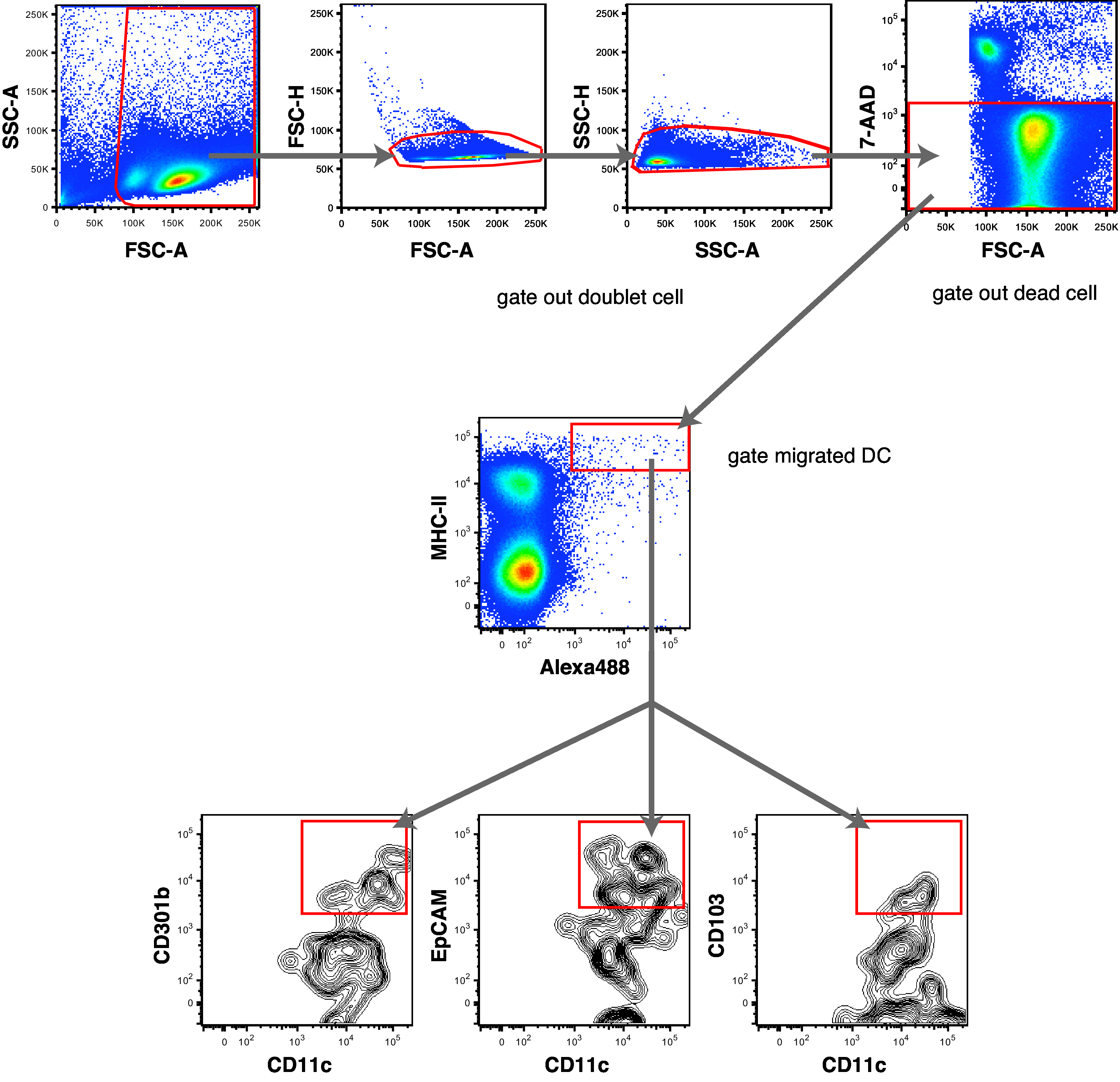

Supplement: S1 Fig — Draining LNs were isolated from mice injected with Alexa488 labeled OVA via the intradermal (ID) or subcutaneous (SC) route and analyzed by flow cytometry. Dot plots show the gating strategy used in this study. DCs that migrated into the draining LN were analyzed on total doublet-, live (7AAD-), Alexa488+, MHC-IIhigh, CD11c+ cells. The three DC subsets were distinguished by surface marker (CD301b, EpCAM, CD103) expression. (TIF) [file pone.0167952.s001.tif]

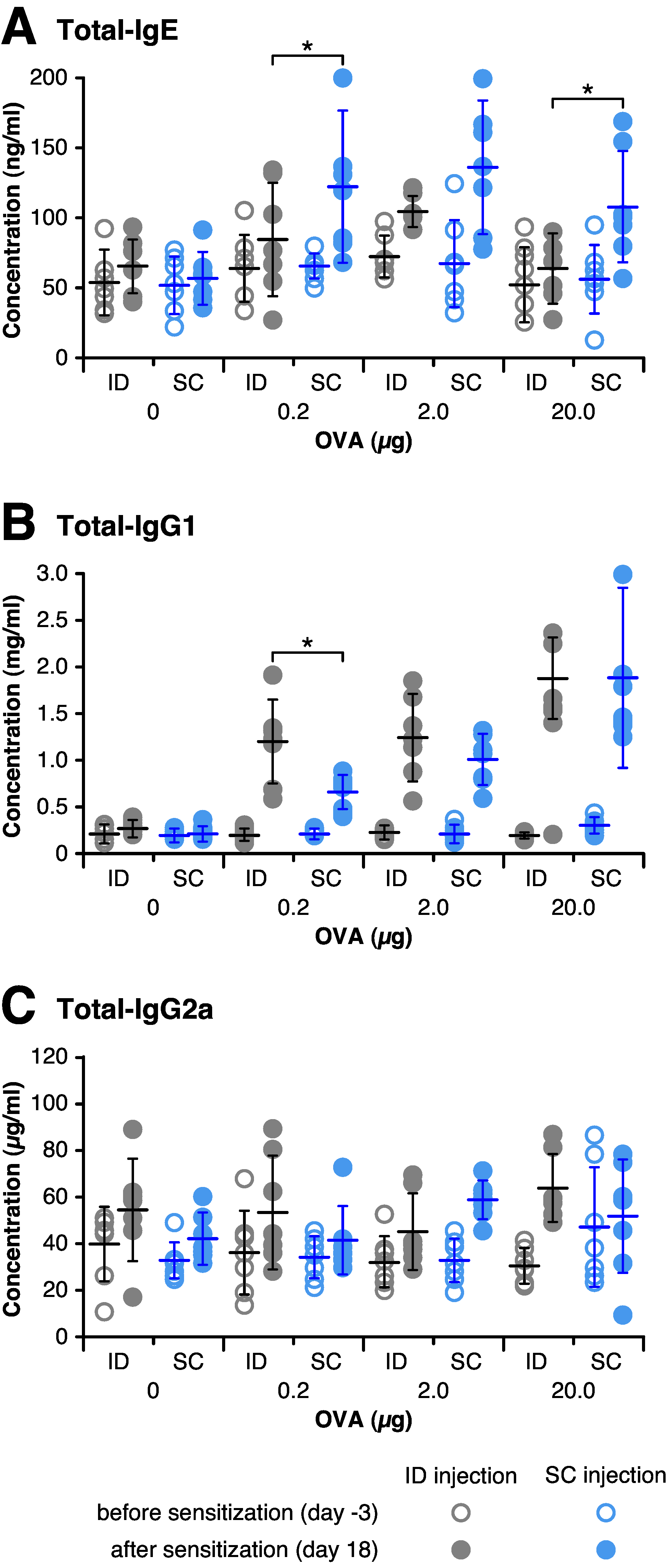

Supplement: S2 Fig — (A–C) BALB/c mice received OVA by intradermal (ID) or subcutaneous (SC) injection. Concentrations of total serum IgE (A), IgG1 (B) and IgG2a (C) were determined by ELISA. Each circle represents the concentration of individual 7 mice, and bar shows the mean ± SD. *P < 0.05, ***P < 0.001. (TIF) [file pone.0167952.s002.tif]

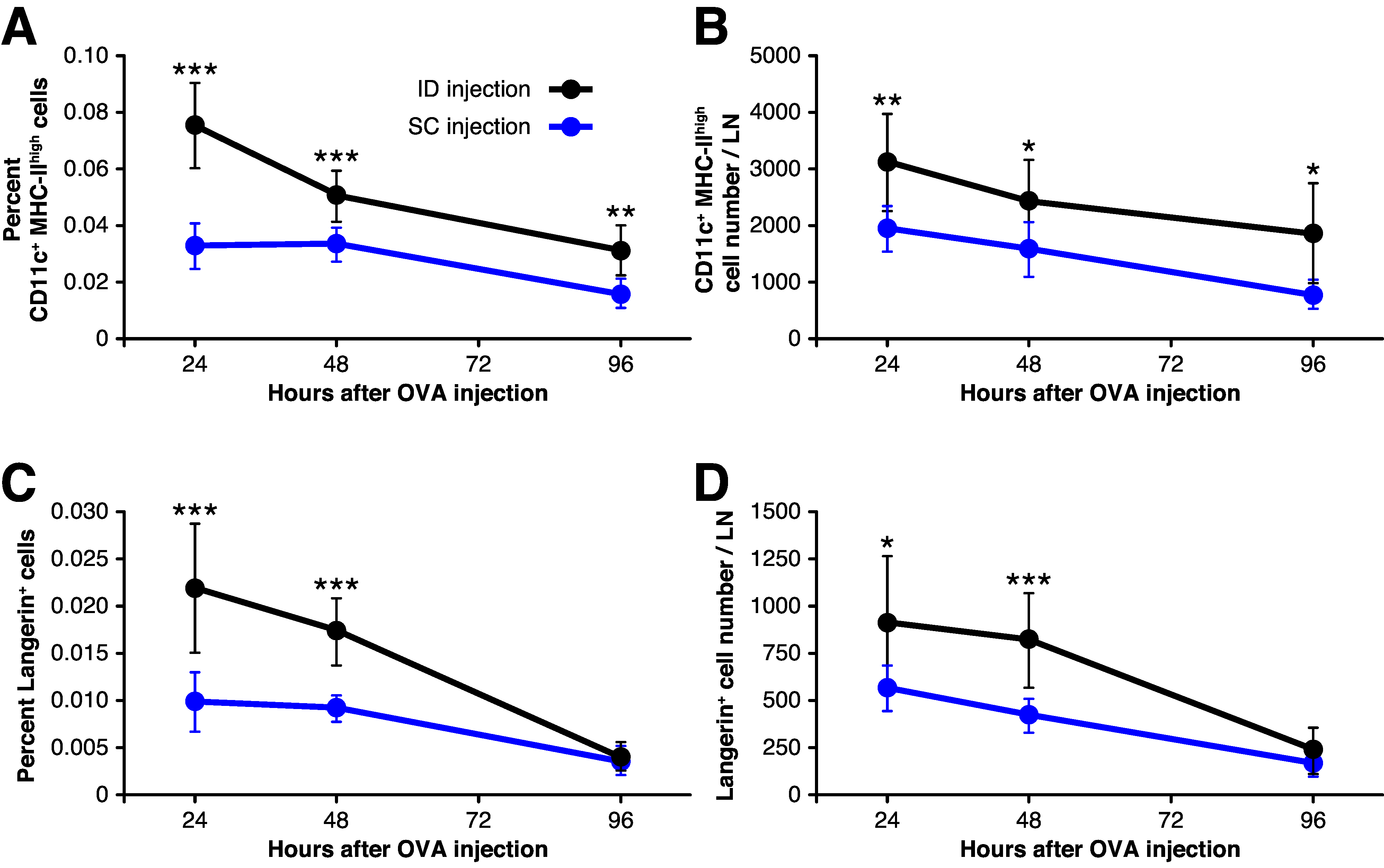

Supplement: S3 Fig — BALB/c mice were injected with OVA via the intradermal (ID) or subcutaneous (SC) route and draining LNs were harvested 24, 48 or 96 hrs after the injection. Cells in draining LNs were analyzed by flow cytometry. (A–D) The percentage (A and C) and absolute number (B and D) of CD11c+MHC-IIhigh cells (A and B) or Langerin+ cells (C and D) within the total doublet-, live cells in the draining LN from ID or SC injected mice. Each bar shows the mean ± SD of 6–8 mice per group. *P < 0.05, **P < 0.01 and ***P < 0.001. (TIF) [file pone.0167952.s003.tif]

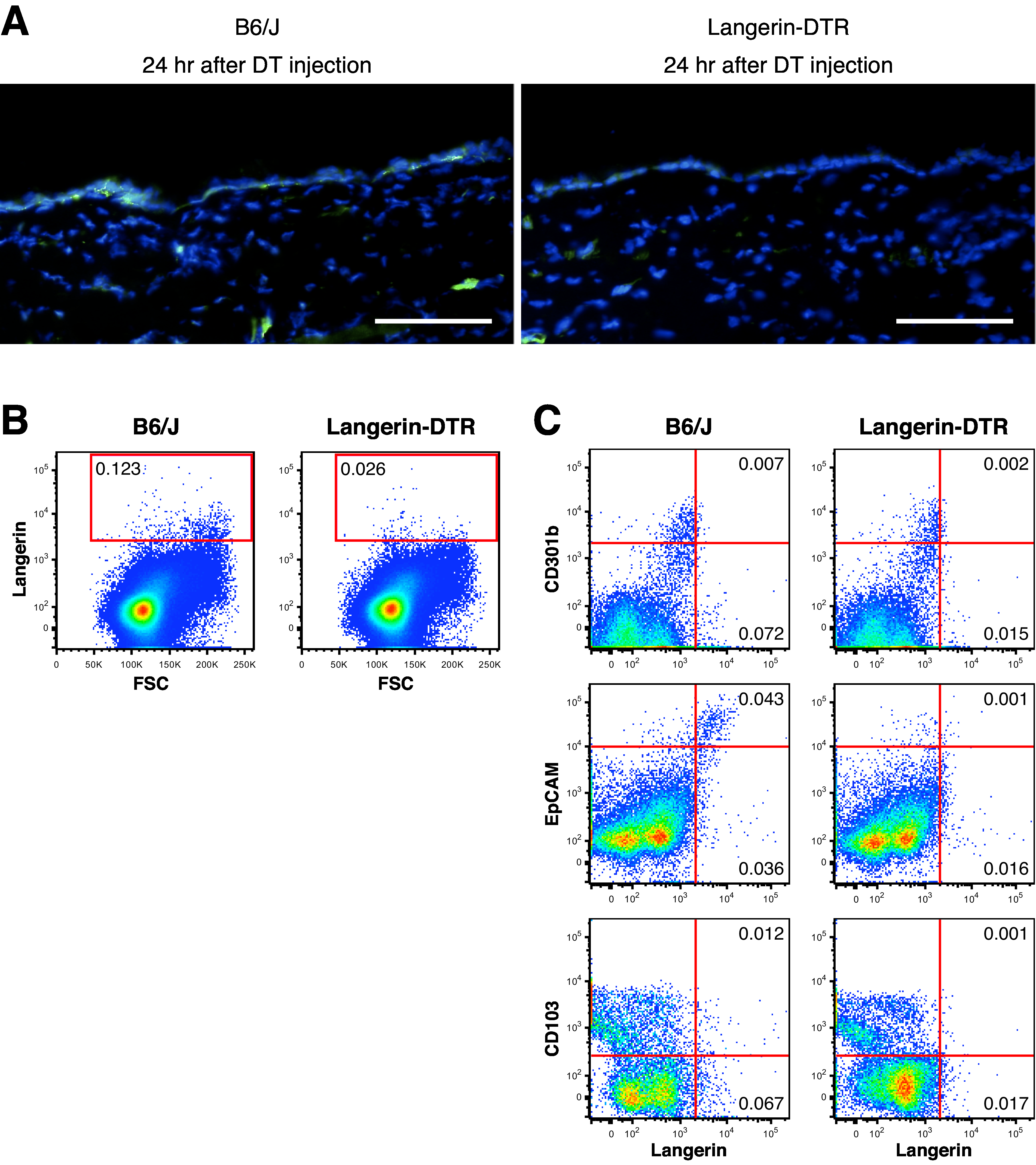

Supplement: S4 Fig — (A) Frozen skin sections of B6/J and Langerin-DTR mice 24 hr after DT injection were stained with anti-Langerin (green), and nuclei were counterstained with DAPI (blue). Scale bar, 100 μm. (B and C) B6/J and Langerin-DTR mice were administered DT via intraperitoneal injection one day before each OVA injection. Mice were injected with OVA via the intradermal (ID) route and draining LNs were harvested 24 hrs later. FACS plots showing cells gated on total LN cells (B) and gated on CD11c+ cells (C) from WT and Langerin-DTR mice. Draining LN cells were stained for the indicated markers. The percentages of Langerin+ cells among total LN cells in each plot are shown. (TIF) [file pone.0167952.s004.tif]

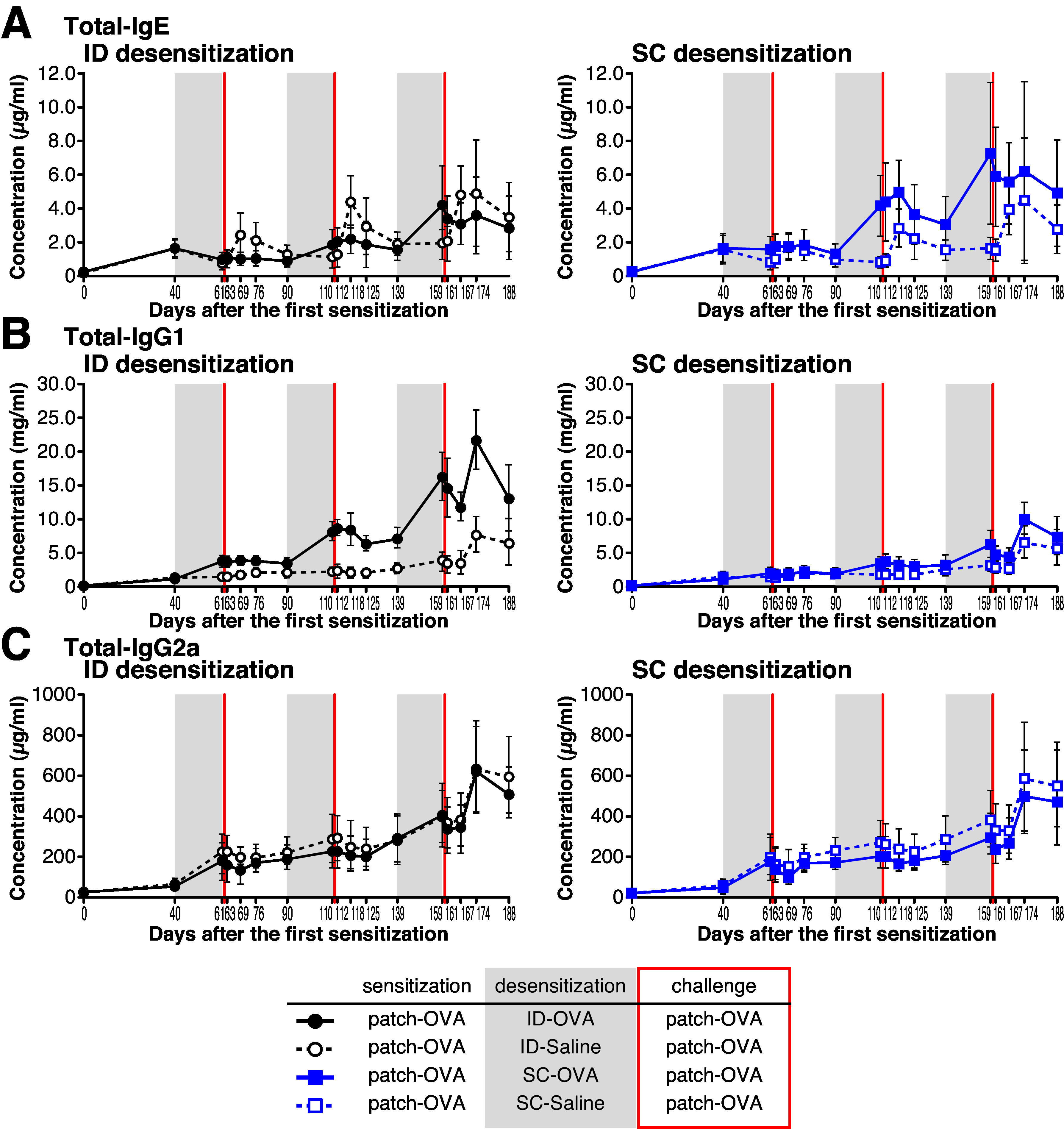

Supplement: S5 Fig — (A–C) Serum concentrations of total IgE (A), IgG1 (B) and IgG2a (C) were determined by ELISA. BALB/c mice were used in this experiment, which was performed as shown in Fig 7A. Data are the mean ± SD of 15 mice per group. (TIF) [file pone.0167952.s005.tif]
